# Severe sepsis and cerebral toxoplasmosis in an HIV-infected late presenter patient

**DOI:** 10.1186/1471-2334-13-S1-P8

**Published:** 2013-12-16

**Authors:** Andreea Ardeleanu, Ovidiu Roşca, Maria Sandu

**Affiliations:** 1 Department of Infectious Diseases I, Dr. Victor Babeş Clinical Hospital of Infectious Diseases and Pneumology, Timişoara, Romania; 2 Department of Infectious Diseases I, Victor Babeş University of Medicine and Pharmacy, Timişoara, Romania; 3 City Hospital, Sânnicolau Mare, Romania

## Background

In Europe, almost a third of individuals infected with HIV do not benefit from healthcare until late in the course of their infection. It is well known that cerebral toxoplasmosis is one of the most common opportunistic neurological infections in AIDS patients, and it is typically observed in the later stages of HIV infection. Moreover, when critically ill HIV patients are admitted to ICUs the main risk factor for hospital mortality is severe sepsis.

## Case report

We report the case of a 56 year-old patient admitted to our department after he had undergone a brain surgery being suspected of a brain tumor. When he was admitted to the neurosurgery department he presented with headache, speaking disorders and progressive hemiplegia. The histopathological examination revealed no malignancies in the brain tissue. Contrast enhanced CT and MRI scans of the head showed a solitary ring-enhanced mass with surrounding edema.

In our department, he presented with high fever, generalized rash, oral candidiasis and right hemiplegia. He tested positive for HIV and serum toxoplasma antibodies. The patient was admitted to the ICU and started anti-toxoplasmosis therapy, but developed severe sepsis with *Acinetobacter baumannii*. The CD4 count was 15 cells/cmm and VL= 756,000 copies/mL.

## Conclusion

Sepsis has a more severe course in HIV-positive patients being the most important risk factor for mortality in HIV/AIDS patients admitted to ICUs. Cerebral toxoplasmosis is still the commonest cerebral opportunistic infection in HIV-infected patients even though the incidence has declined with the use of antiretroviral therapy. It is often diagnosed as an initial presentation of HIV infection affecting short- and longer-term survival.

